# Supplementary material for: Intratracheal Transplantation of Mesenchymal Stem Cells Attenuates Hyperoxia-Induced Microbial Dysbiosis in the Lungs, Brain, and Gut in Newborn Rats
Source: Int J Mol Sci. 2022 Jun 13;23(12):6601. doi: 10.3390/ijms23126601 (PMC9223745; doi:10.3390/ijms23126601)
Supplement: Supplementary file 1 [file ijms-23-06601-s001.zip › ijms-1699866-supplementary.pdf]

Supplementary Figure S1

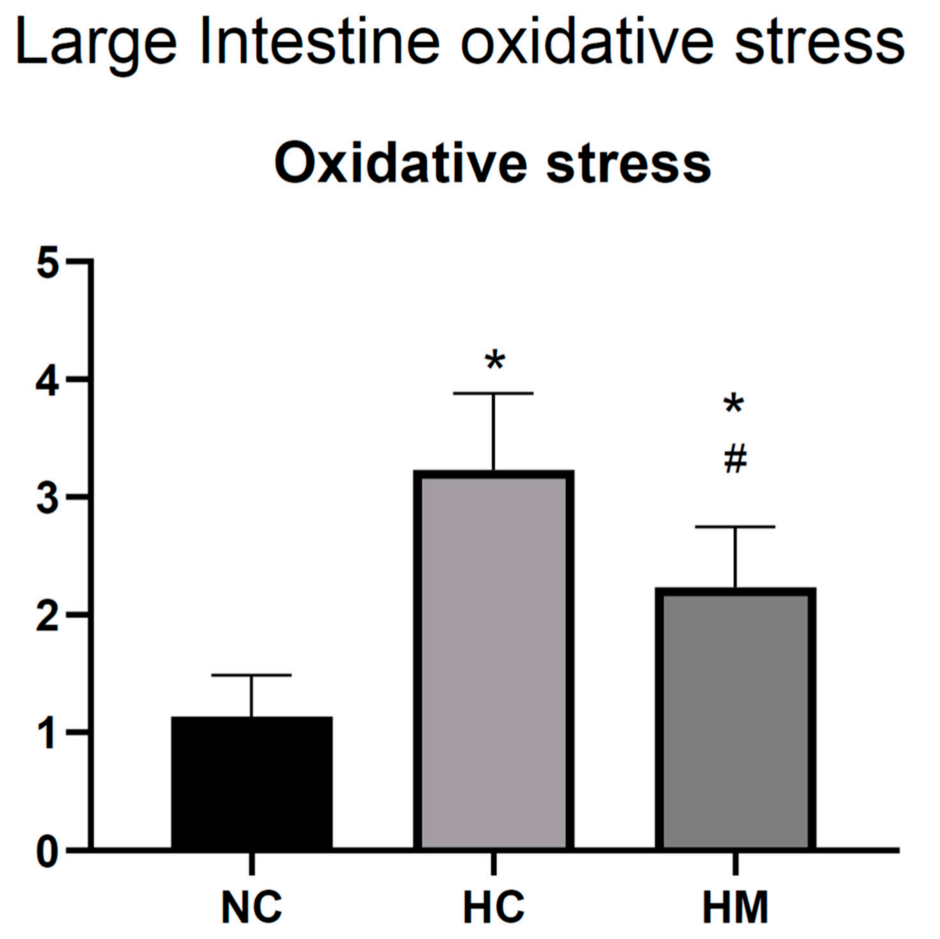

Figure S1. Large intestine oxidative stress.

Oxidative stress was assessed with levels of Malondialdehyde in the large intestine in each group. Data are presented as the mean ± standard deviation. Abbreviations: NC, normoxia control; HC, hyperoxia control; HM, hyperoxia with transplantation of human UCB-MSCs.
*, P < 0.05 compared with the NC group; #, P < 0.05 compared with the HC group.
